# Machine learning approach to support taxonomic species discrimination based on helminth collections data

**DOI:** 10.1186/s13071-021-04721-6

**Published:** 2021-05-01

**Authors:** Victor Hugo Borba, Coralie Martin, José Roberto Machado-Silva, Samanta C. C. Xavier, Flávio L. de Mello, Alena Mayo Iñiguez

**Affiliations:** 1 Laboratório de Biologia de Tripanosomatídeos-LABTRIP, Instituto Oswaldo Cruz, IOC-FIOCRUZ, Rio de Janeiro, RJ Brazil; 2 Laboratório de Helmintologia Romero Lascasas Porto, Faculdade de Ciências Médicas, UERJ, Rio de Janeiro, RJ Brazil; 3 Unité Molécules de Communication et Adaptation des Microorganismes (MCAM, UMR 7245), Muséum National d’Histoire Naturelle, CNRS, CP52, Paris, France; 4 Departamento de Engenheira Eletrônica e Computação, Universidade Federal do Rio de Janeiro, Rio de Janeiro, RJ Brazil

**Keywords:** Taxonomy, Artificial intelligence, Species identification, Capillaridae, Parasite eggs

## Abstract

**Background:**

There are more than 300 species of capillariids that parasitize various vertebrate groups worldwide. Species identification is hindered because of the few taxonomically informative structures available, making the task laborious and genus definition controversial. Thus, its taxonomy is one of the most complex among Nematoda. Eggs are the parasitic structures most viewed in coprological analysis in both modern and ancient samples; consequently, their presence is indicative of positive diagnosis for infection. The structure of the egg could play a role in genera or species discrimination. Institutional biological collections are taxonomic repositories of specimens described and strictly identified by systematics specialists.

**Methods:**

The present work aims to characterize eggs of capillariid species deposited in institutional helminth collections and to process the morphological, morphometric and ecological data using machine learning (ML) as a new approach for taxonomic identification. Specimens of 28 species and 8 genera deposited at Coleção Helmintológica do Instituto Oswaldo Cruz (CHIOC, IOC/FIOCRUZ/Brazil) and Collection de Nématodes Zooparasites du Muséum National d’Histoire Naturelle de Paris (MNHN/France) were examined under light microscopy. In the morphological and morphometric analyses (MM), the total length and width of eggs as well as plugs and shell thickness were considered. In addition, eggshell ornamentations and ecological parameters of the geographical location (GL) and host (H) were included.

**Results:**

The performance of the logistic model tree (LMT) algorithm showed the highest values in all metrics compared with the other algorithms. Algorithm J48 produced the most reliable decision tree for species identification alongside REPTree. The Majority Voting algorithm showed high metric values, but the combined classifiers did not attenuate the errors revealed in each algorithm alone. The statistical evaluation of the dataset indicated a significant difference between trees, with GL + H + MM and MM only with the best scores.

**Conclusions:**

The present research proposed a novel procedure for taxonomic species identification, integrating data from centenary biological collections and the logic of artificial intelligence techniques. This study will support future research on taxonomic identification and diagnosis of both modern and archaeological capillariids.

**Graphical abstract:**

**Supplementary Information:**

The online version contains supplementary material available at 10.1186/s13071-021-04721-6.

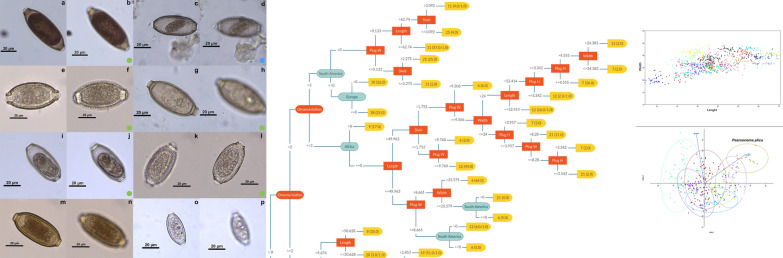

## Background

There are more than 300 species of capillariids that parasitize various vertebrate groups (fish, amphibians, reptiles, avian and mammals) worldwide [1]. Species identification is hindered because of the few taxonomically informative structures available, making the task laborious and the genus or species definition controversial. Consequently, its taxonomy is one of the most complex among Nematoda, which makes the identification at the genus or species level difficult.

Moravec (1982) proposed a new taxonomy classification for capillariids to serve as a foundation for future studies, thus raising the genera to family Capillaridae Neveu-Lemaire, 1936 (Nematoda: Trichocephalida), because of the difference in worm morphologies, the variety of infection sites and their definitive hosts. The taxonomy of the genera was based mainly on morphological characteristics of the posterior termination of males. Therefore, dividing the capillariids into 16 genera (12 redefined, 2 rescued and 2 created) was suggested [1].

The suggested genera were: *Schulmanela* Ivashkin, 1964, *Paracapillaria* Mendonça, 1963, *Capillostrongyloides* Freitas and Lent, 1935; *Pseudocapillaria* Freitas, 1959; *Liniscus* Dujardin, 1845; *Pearsonema* Freitas and Mendonça, 1960; *Echinocoleus* López-Neyra, 1947; *Capillaria* Zeder, 1800; *Eucoleus* Dujardin, 1845; *Pterothominx* Freitas, 1959; *Aonchotheca* López-Neyra, 1947; *Calodium* Dujardin, 1845; *Gessyella* Freitas, 1959; *Skrjabinokillaria* Skarbilovich, 1946. Additionally, two new genera were described, *Freitascapillaria* gen. n. and *Baruscapillaria* gen. n. [1]. Subsequently, other genera were added to the family, totaling 22 genera. These are: *Pseudocapillaroides* Moravec and Cosgrove, 1982; *Piscicapillaria* Moravec, 1982; *Amphibiocapillaria* Moravec, 1982; *Tenoranema* Mas-Coma and Esteban, 1985; *Paratrichosoma* Ashford and Muller, 1978 [2].

In 2010, Gibbons expanded the classification proposing other genera in the subfamily Capillarinae. Some of the genera that were classified in this subfamily are: *Tridentocapillaria* Barus and Sergeeva, 1990; *Brevithominx* Teixeira de Freitas and Machado de Mendonça, 1964; *Paracapillaroides* Moravec, Salgado-Maldonado and Caspeta-Mandujano, 1999; *Crocodylocapillaria* Moravec and Spratt, 1998 [3]. Although scarce, some molecular studies were performed to support the systematic classification of the group and confirmed the classification of the genera proposed by Moravec (1982) [4–6].

Eggs are the parasitic structures most viewed in coprological analysis, both in modern samples, from public health or ecological surveys, and in ancient samples, from paleoparasitological studies [7]. Most of the eggs detected in ancient samples are not identified at the genus or species level, and in modern samples, when just eggs are detected, the identification is impaired [6]. Although species and genera of capillariids are identified primarily based on the structure of the posterior end of male adults, the structure of the egg could also play a role in genera or species discrimination [1, 8].

Artificial intelligence (AI) is described as the ability of a machine to perform “intelligent” functions, for instance, learning, decision-making, adaptation, control and perception [9]. To execute such functions, a classification process must be triggered so that scenarios can be identified, grouped and properly treated. Machine learning (ML) is a useful AI approach when this classification process depends on a huge data analysis. ML has been used for epidemiological research [10], diagnosis [11], discriminating pathogens [12] and for resolving taxonomic relationships with molecular data [13]. Thus, we propose that the complexity of Capillariidae species definition, based on egg structures, could be clarified using AI tools. A taxonomic dataset including morphological and morphometrical characteristics of parasite eggs and ecological information was constructed based on specimens from institutional helminth collections. Institutional biological collections are taxonomic respositories of specimens described and strictly identified by experienced taxonomists. The current research proposed a novel procedure for taxonomic species identification, integrating data from centenary biological collections and the logic of artificial intelligence approaches.

## Materials and methods

### Morphological and morphometric analyses

The specimens were collected from two institutional helminth collections: the Coleção Helmintológica do Instituto Oswaldo Cruz (CHIOC) from Fundação Oswaldo Cruz (FIOCRUZ), Brazil, 14 species (20 specimens), and the Collection de Nématodes Zooparasites du Muséum National d’Histoire Naturelle de Paris (MNHN), France, 16 species (17 specimens).

The eggs were separated from the specimens for morphological and morphometric analyses. Females containing eggs were collected to separate eggs or fragments containing eggs when it was not possible to manually extract them from inside the females. Eggs were extracted from the final portion of the uterus. For clear visualization of egg morphometry, samples were subjected to an ultrasonic bath (Cristófoli^®^) for 60 s at the frequency of 42 hHz. The process was done to clean dirt and fragments from females, so that only eggs with the chitin shell were present.

**Fig. 1:**
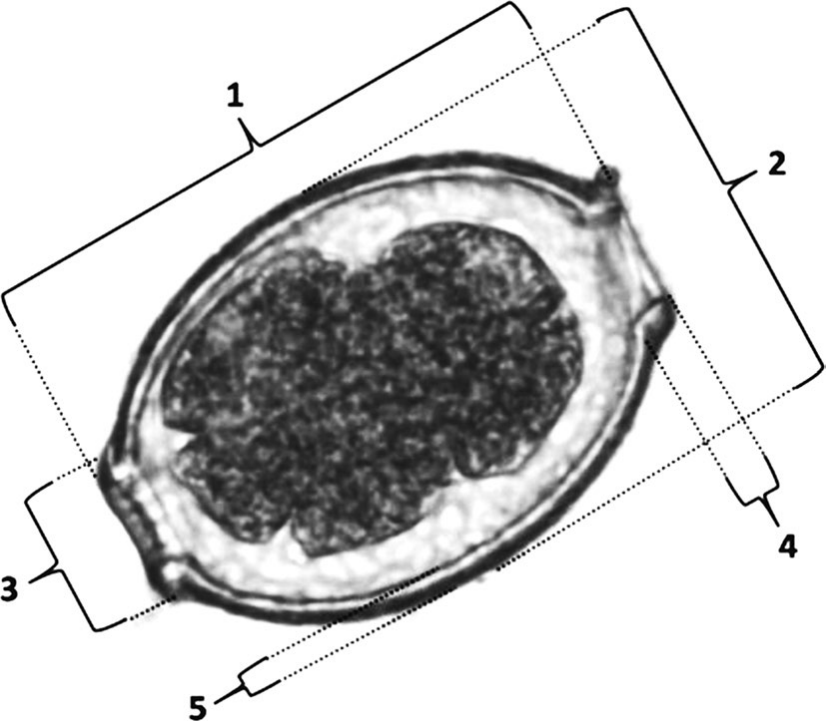
Representation of capillariid egg measurements considered in the present study based on *Baruscapillaria obsignata* voucher CHIOC26715. The five measures taken were: 1: total length; 2: total width; 3: base of the polar plug width; 4: base of the polar plug height; 5: shell thickness

**Fig. 2:**
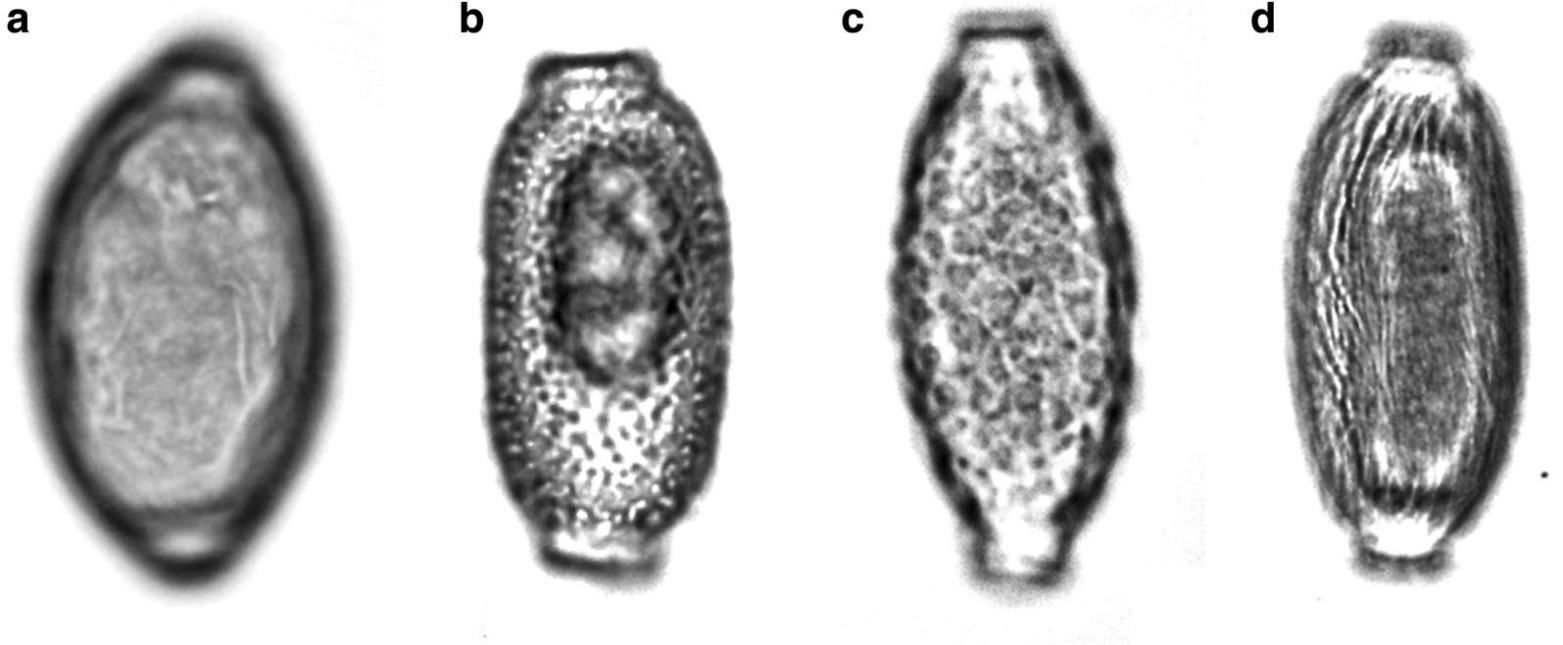
Representation of each ornamentation pattern considered in the present study based on capillariid eggs from CHIOC species. **a** Smooth from *Aonchotheca pulchra* voucher CHIOC9804; **b** punctuated from *Capillaria brasiliana* voucher CHIOC7046; **c** reticulated type I from *Pearsonema plica* voucher MNHN373; **d** reticulated type II from *Baruscapillaria resecta* voucher MNHN1073. Representations are based on images that are intentionally blurry on the general plane to focus on the ornamentation plane

The eggs' morphology and morphometry were characterized by an optical microscope (Nikon Eclipse E200) at 400× magnification using image analysis software (Image Pro Plus—Media Cybernetics, USA). Thirty eggs per specimen were evaluated, whenever available. The measures considered were: total diameter (width) and length of the eggs, mean value of the width and height of the two plugs and the thickness of the shell (Fig. 1). A qualification of the ornaments presented in the outer bark of capillariid eggs was also performed. The parameter of egg ornamentation was divided in four categories following the literature [14]: (1) smooth, which has no ornaments on the shell, as described by Conboy for *Trichuris trichiura* eggs [15]; (2) punctuated, which has dots like a pitted surface, as described in *Eucoleus bohemi* by Conboy and Traversa et al. [15, 16]; (3) reticulated type I (RTI), which presents like a network of interconnected ridges as described in *Eucoleus aerophilus* by Conboy [15]; (4) reticulated type II (RTII), which presents like a network but with an orientation of deep longitudinal ridges, as described in *Aonchotheca putorii* by Zajac and Conboy [17] (Fig. 2a–d).

### Discriminant analyses and artificial intelligence/machine learning approaches

A dataset of capillariid species from FIOCRUZ and MNHN collections was constructed with the morphological (eggshell ornamentation) and morphometric parameters (MM) (total length and width, base of the polar plug width and height and shell thickness) generated by specimens. In addition, ecological parameters, such as information about the host (H) and geographical location (GL) of specimens, were included. A total of 997 entries were generated (Additional file 1: Table S1).

Discriminant analyses were performed using Past 3.16 software to separate species groups. First, the total length and width of eggs from all species were plotted; then, the discriminant function analysis was generated by each eggshell ornamentation: punctual, RTI and RTII. The exception was smooth ornamentation with only one species identified.

For ML/AI analyses, ornamentation and ecological parameters were encoded into numerical variables. Ecological parameters were defined as host (fish, amphibian, reptile, avian, mammal) and as geographical location (South America, Central America, North America, Europe, Africa, Asia, Oceania). Response variables were 1 = yes or presence; 0 = no or absence; − 1 = no information available. To evaluate the more reliable set of sample information to lead to an identification, MM parameters were tested alone and in combination with ecological parameters, MM + H, MM + GL and MM + H + GL.

Since no literature on ML algorithms is applied to taxonomic species definition, an exhaustive test of several algorithms available on Weka 3.8.3 software [18] was conducted. In addition, the present research looked for new criteria to find, describe and name particular species, while keeping the top-down approach of a taxonomy rank. There are several ML/AI algorithms for classification, but only some of them provide decision trees which are similar to the taxonomic keys proposed/used by systematics specialists to discriminate biological species. Therefore, we focused on Weka’s algorithms, which returned representations of decision trees, namely: J48 [19], Random Tree [20], REPTree [21] and Logistic Model Tree (LMT) [22]. The ML classification algorithms produced training models that were tested using cross-validation, providing kappa values. Moreover, we implemented an additional classification using a Majority Voting algorithm [23], which integrates all four decision tree classifiers to combine the predictions from multiple ML algorithms and to exploit the different peculiarities of each algorithm. The performance of five algorithms was reported as metrics of sensitivity, specificity, negative predictive value (NPV), positive predictive value (PPV) and accuracy [24] in addition to the correct instances percentage, kappa coefficient and area under the receiver-operating characteristic (ROC) curve (AUC), as informed by Weka.

Statistical analysis was applied to check the null hypothesis for equal proportions of the AUC values among the algorithms—J48, Random Tree, REPTree, Logistic Model Tree and Majority Voting (H0 p1 = p2 = p3 = p4 = p5)—and among parameters—MM + H + GL, MM + H, MM + GL and MM + H + GL (H0 p1 = p2 = p3 = p4). To arrive at a conclusion about the hypothesis with 95% confidence, the *P*-value of the chi-square statistic should be < 0.05, indicating that the difference is significant, and < 0.01 for highly significant. Subsequently, the Marascuilo procedure was applied to check which proportions were different among the algorithms and among the combinations of parameters applied. Data analyses were performed using RStudio version 3.5.1 (2018-07-02) software.

## Results

### Morphological and morphometric analyses

**Fig. 3:**
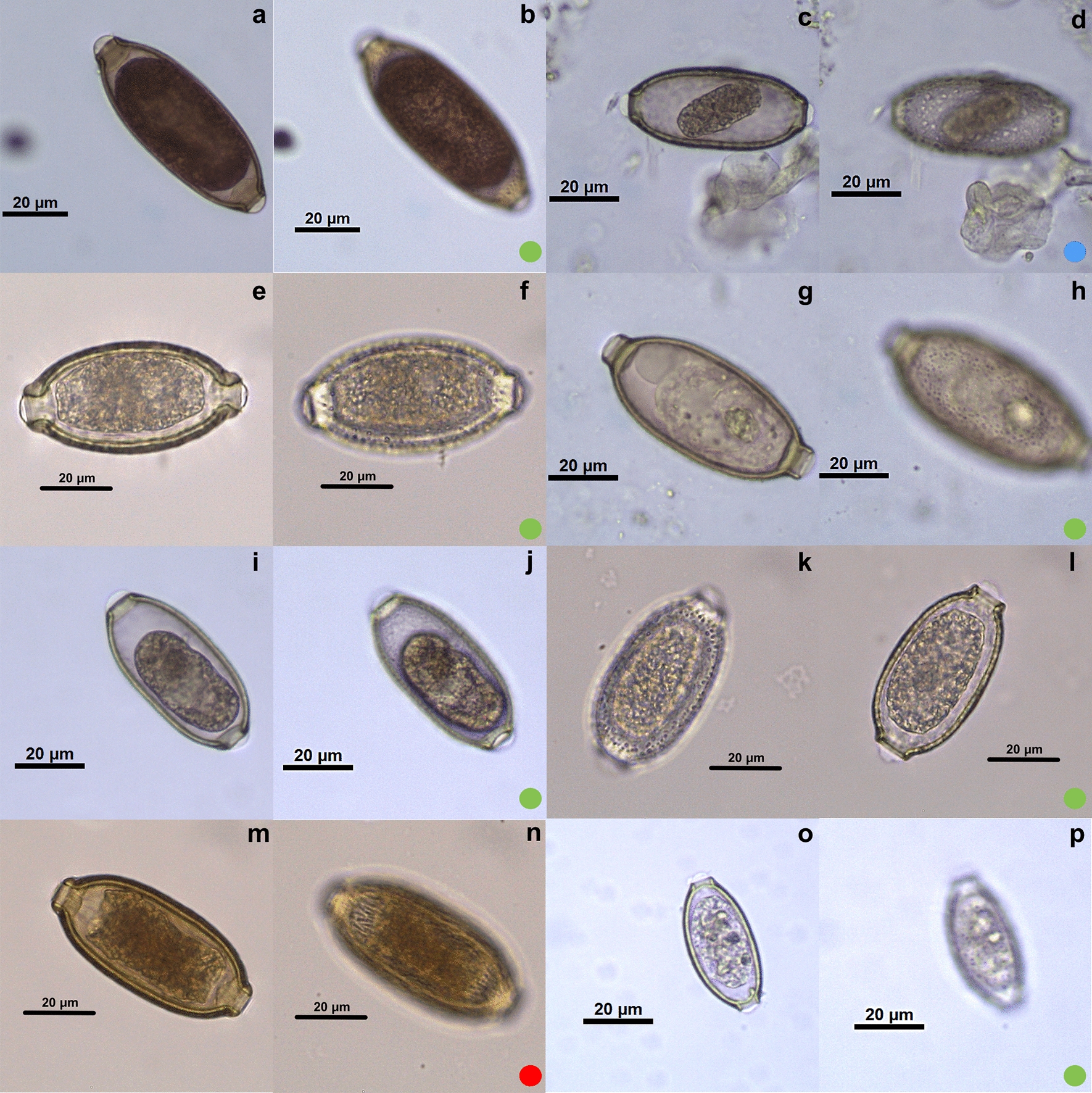
Micrographies of *Eucoleus* genus eggs. The first image (**a**, **c**, **e**, **g**, **i**, **k**, **m**, **o**) of each species is an egg overview, and the second image (**b**, **d**, **f**, **h**, **j**, **l**, **n**, **p**) focuses on ornamentation. **a**, **b** *Eucoleus anullatus*; **c**, **d** *E. dubius*; **e**, **f** *E. bacilatus*; **g**, **h** *E. eberthi*; **i**, **j** *E. contortu*; **k**, **l** *E. madjerdae*; **m**, **n** *E. dispar*; **o**, **p** *E. perforans*. Each colored dot represents an ornamentation pattern: green dot: punctuated; blue dot: reticulated type I; red dot: reticulated type II. Images intentionally focus on the ornamentation plane

**Fig. 4:**
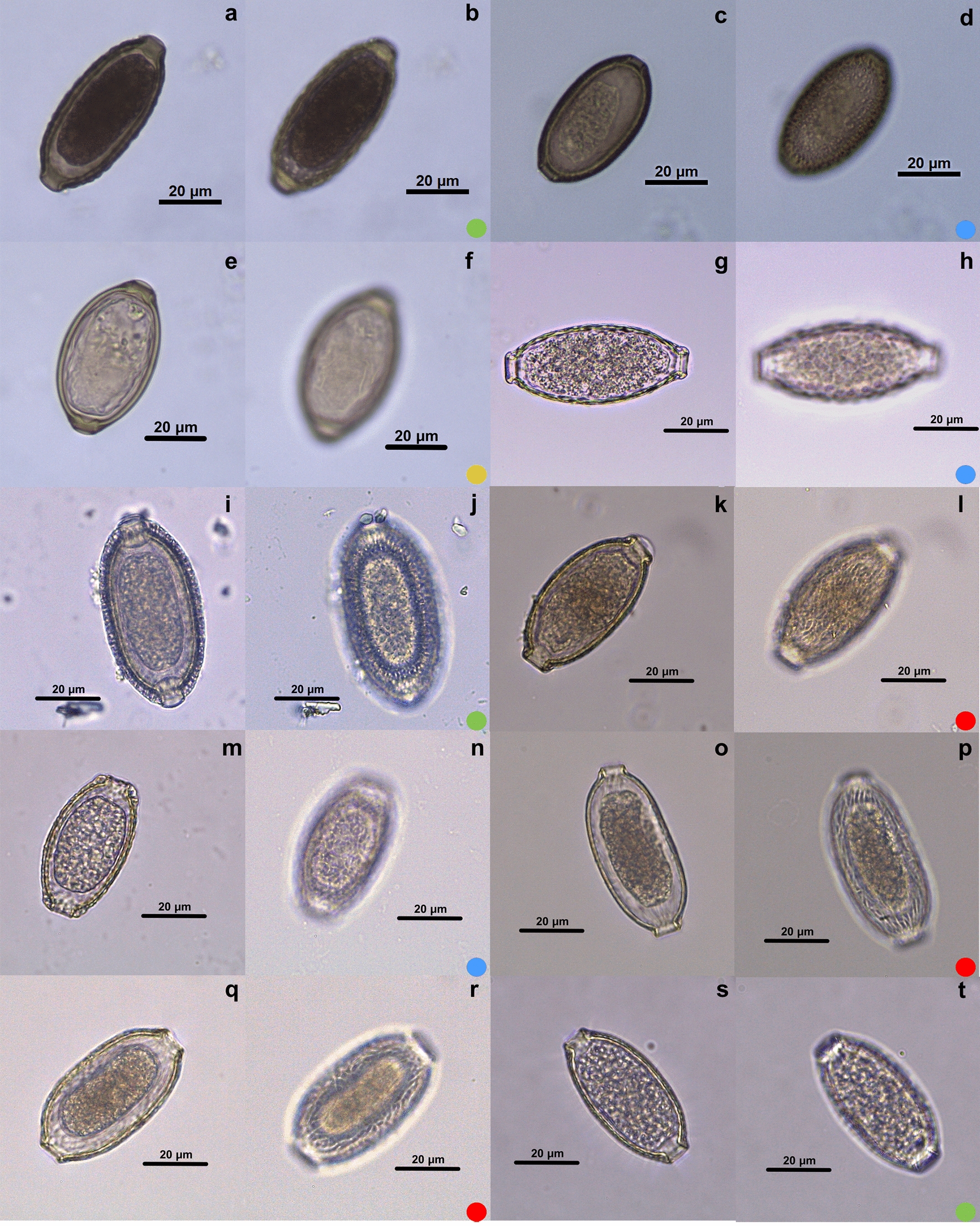
Micrographies of eggs belonging to *Echinocoleus, Pterothominx, Pearsonema, Calodium* and *Aonchotheca* genera. The first image (**a**, **c**, **e**, **g**, **i**, **k**, **m**, **o**) of each species is an egg overview, and the second image (**b**, **d**, **f**, **h**, **j**, **l**, **n**, **p**) focuses on ornamentation. **a**, **b** *Echinocoleus auritae*; **c**, **d** *E. hydrocoeri*; **e**, **f** *P. pulchra*; **g**, **h** *P. plica*; **i**, **j** *C. hepaticum*; **k**, **l** *A. annulosa*; **m**, **n** *A. baylisi*; **o**, **p** *A. myoxinitelae*; **q**, **r** *A. erinaceid*; **s**, **t** *A. murissylvatici*. Each colored dot represents an ornamentation pattern: yellow dot: smooth; green dot: punctuated; blue dot: reticulated type I; red dot: reticulated type II. Images intentionally focus on the ornamentation plane

**Fig. 5:**
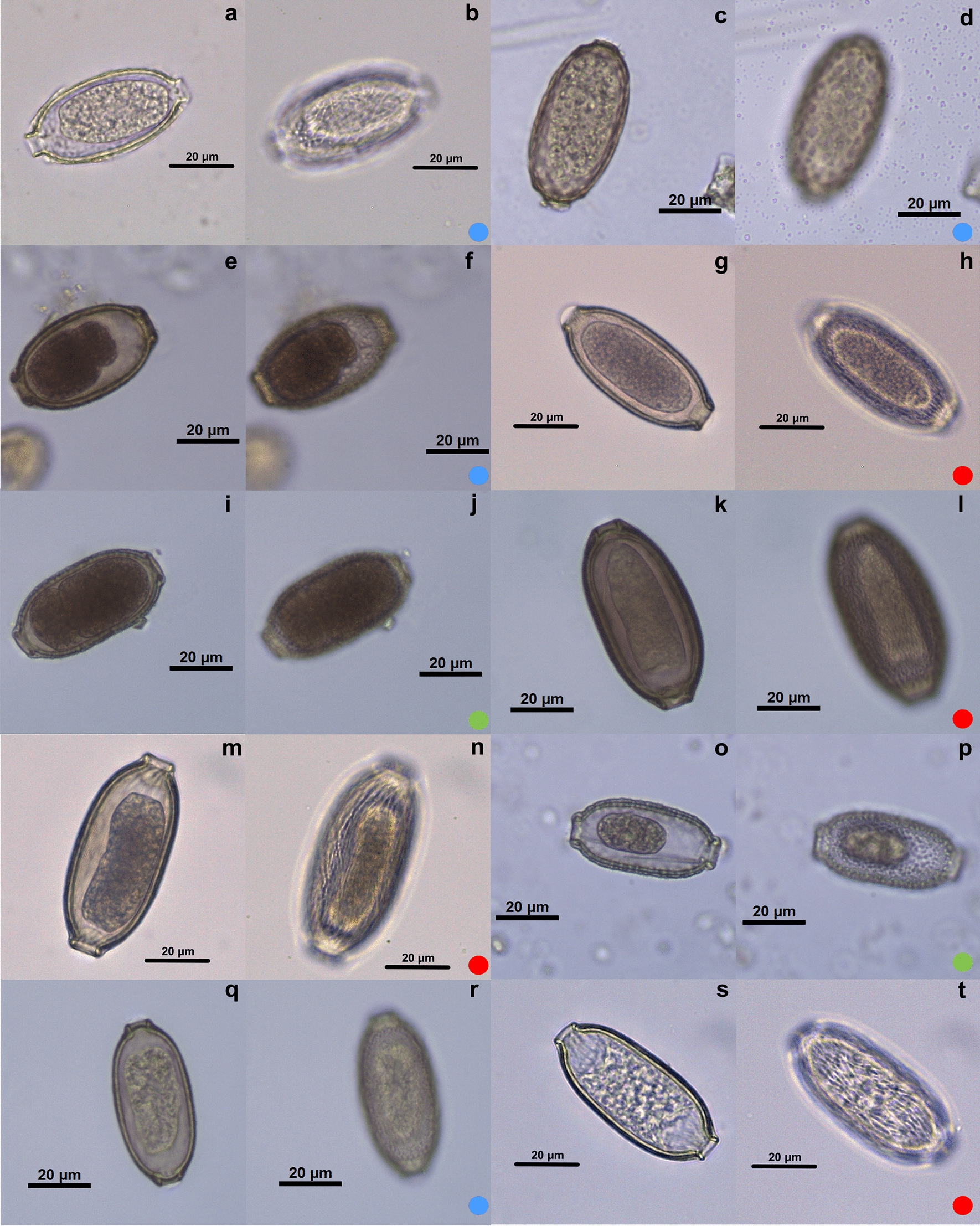
Micrographies of eggs belonging to *Baruscapillaria, Capillaria* and *Tridentocapillaria* genera. The first image (**a**, **c**, **e**, **g**, **i**, **k**, **m**, **o**) of each species is an egg overview, and the second image (**b**, **d**, **f**, **h**, **j**, **l**, **n**, **p**) focuses on ornamentation. **a**, **b** *Baruscapillaria falconis*; **c**, **d** *Capillaria collaris*; **e**, **f** *B. obsignata*; **g**, **h** *C. exigua*; **i**, **j** *B. spiculata*; **k**, **l** *C. venusta*; **m**, **n** *B. resecta*; **o**, **p** *C. brasiliana*; **q**, **r** *B. rudolphi*; **s**, **t** *Tridentocapillaria tridens*. Each colored dot represents an ornamentation pattern: green dot: punctuated; blue dot: reticulated type I; red dot: reticulated type II. Images intentionally focus on the ornamentation plane

The species of Capillariidae studied here, in general, presented a barrel shape, varying between round and elongated, with polar plugs, and the eggshell usually had ornamentation, as described in the literature [25]. A total of 28 species of capillariids distributed in eight genera were characterized. Regarding eggshell ornaments, they were classified as smooth (*n* = 1), punctuated (*n* = 10), RTI (*n* = 7) and RTII (*n* = 10) (Figs.  3, 4, 5; Table 1) (*n* = number of species classified in each ornamentation).

**Table 1:** Morphometry of Capillariidae species with measurements of length, width, plug width, plug thickness and shell thickness

Species	Length (μm)		Width (μm)		Plug base W (μm)		Plug base H (μm)		Shell (μm)		Shell ornamentation
	Mean	Amplitude	Mean	Amplitude	Mean	Amplitude	Mean	Amplitude	Mean	Amplitude	
*Aonchotheca annulosa*	53.11	49.28–55.96	28.04	26.03–32.60	8.91	7.23–10.28	3.50	2.52–4.58	2.66	1.98–3.36	4
*Aonchotheca baylisi*	46.56	44.75–50.14	26.50	24.92–28.97	7.75	6.40–9.28	3.85	2.69–5.27	2.66	1.78–3.48	4
*Aonchotheca erinacei*	54.81	52.67–57.37	30.98	27.99–33.70	9.66	7.99–12.26	3.00	1.83–4.04	2.50	1.64–3.09	4
*Aonchotheca murissylvatici*	53.10	50.75–55.70	26.17	24.91–27.82	8.43	7.33–9.74	3.74	2.75–4.83	2.80	2.29–3.42	2
*Aonchotheca myoxinitelae*	57.74	55.44–61.57	25.83	24.77–26.76	8.25	6.83–9.24	3.21	2.47–4.06	1.68	1.34–2.11	4
*Aonchotheca pulchra*	49.52	46.15–52.96	30.89	28.15–34.40	8.52	7.42–9.38	4.09	2.77–5.68	1.82	1.41–2.32	1
*Baruscapillaria obsignata*	47.46	42.17–51.78	27.94	24.76–33.45	9.97	7.13–12.94	2.84	1.09–4.62	1.77	1.18–2.52	3
*Baruscapillaria rudolphi*	54.34	52.57–57.56	24.84	22.88–26.84	8.33	6.74–9.37	4.01	3.34–4.85	2.18	1.78–2.61	3
*Baruscapillaria spiculata*	53.17	51.48–55.69	27.02	24.11–30.47	1.85	9.17–12.01	2.67	1.98–3.61	2.38	1.88–2.79	2
*Baruscapillaria falconis*	54.30	52.54–55.85	26.23	25.28–28.19	7.95	6.92–8.68	3.32	2.51–4.06	1.51	1.00–2.54	4
*Baruscapillaria resecta*	68.12	65.47–70.39	30.53	29.58–31.81	9.56	8.37–10.79	3.88	2.87–5.07	2.73	1.85–3.43	4
*Capillaria venusta*	60.21	54.01–63.29	30.00	21.95–32.27	9.95	8.56–11.65	3.60	2.54–4.60	2.83	1.45–3.98	4
*Capillaria colaris*	52.33	46.91–56.82	26.46	23.81–30.03	8.32	7.24–9.04	3.36	2.20–4.64	1.46	0.84–2.22	3
*Capillaria brasiliana*	46.45	43.10–50.24	21.41	19.06–22.88	8.80	7.23–10.66	2.67	1.66–3.6	2.05	1.63–2.86	2
*Capillaria exigua*	55.10	52.73–56.73	26.33	25.18–27.12	8.06	7.13–9.08	2.84	1.94–3.59	1.61	1.31–1.98	4
*Calodium hepaticum*	55.44	50.07–62.02	30.42	27.38–33.84	8.08	7.07–9.86	4.24	3.16–5.18	4.34	3.26–5.57	2
*Echinocholeus hydrochoeri*	49.34	46.18–51.74	25.14	22.43–27.62	6.72	5.92–7.91	3.78	2.58–5.00	2.65	1.59–3.63	2
*Echinocholeus auritae*	57.69	56.13–59.84	26.13	24.71–27.82	7.74	6.85–8.80	4.84	3.99–6.15	2.72	2.1–3.51	3
*Eucoleus perforans*	39.86	37.06–42.81	20.41	18.15–23.94	6.37	5.43–7.45	2.50	2.05–3.38	1.57	1.08–2.24	2
*Eucoleus annulatus*	65.36	61.45–68.78	26.75	24.87–27.71	8.56	6.24–10.22	3.33	2.47–5.33	1.13	0.78–1.63	2
*Eucoleus contortus*	51.07	46.70–54.07	26.24	24.49–28.21	8.03	6.71–9.62	2.36	1.59–3.77	1.42	1.08–1.82	2
*Eucoleus dubius*	52.14	47.07–55.25	23.62	22.40–25.57	8.82	7.73–9.80	3.43	2.44–4.24	2.28	1.76–2.73	3
*Eucoleus eberthi*	65.89	63.15–69.82	29.27	28.55–29.86	9.59	8.53–10.55	5.30	4.23–7.01	1.59	1.25–1.97	2
*Eucoleus bacilatus*	63.09	60.53–68.29	32.78	32.04–33.65	11.24	9.70–12.95	4.78	3.86–6.05	3.97	3.17–4.63	2
*Eucoleus madjerdae*	53.41	51.81–55.10	29.23	28.41–29.99	10.38	8.82–11.19	3.29	2.16–4.53	2.13	1.53–2.53	2
*Eucoleus dispar*	63.42	60.34–68.39	29.65	28.19–32.81	8.89	7.63–10.93	3.94	2.79–5.40	2.90	2.34–3.68	4
*Pearsonema plica*	62.62	60.30–65.32	27.47	26.35–28.76	9.62	8.48–10.87	4.47	3.40–5.28	2.24	1.71–2.55	3
*Tridentocapillaria tridens*	60.60	57.39–63.33	27.54	25.84–29.81	8.34	7.04–9.23	2.84	1.99–4.22	2.86	2.49–3.13	4

Shell ornamentations: 1, smooth; 2, punctuated; 3, reticulated type I; 4, reticulated type II

In all genera with more than one species to compare, a high heterogeneity of measurements was observed: an amplitude of 37.06–70.39 μm for length, 18.15–34.40 μm for width, 5.43–12.95 μm for plug base width, 1.09–5.68 μm for plug base height and 0.78–5.57 μm for eggshell thickness (Table 1; Additional file 1: Table S1).

### *Genus *Aonchotheca

Five species were collected from *Collection de Nématodes Zooparasites* of MNHN. The hosts of all the species were registered as mammals: *Aonchotheca annulosa* in *Apodemus sylvaticus*; *A. baylisi* in *Lophuromys sikapusi*; *A. erinaceid* in *Erinaceus europaeus*; *A. murissylvatici* in *Evotomys glareolus*; *A. myoxinitelae* in *Eliomys quercinus; A. pulchra* in *Tadarida laticaudata* and *Nyctinomus brasiliensis*. In general, the egg morphology was very similar, and the plug bases were mostly prominent, except in *A. baylisi*, which had a thickening of the eggshell in the plug base region, masking the prominence. The most common egg ornamentation was RTII with four species (Fig. 5l, n, p, r). One punctuated type ornamentation was present (Fig. 5t). *Aonchotheca pulchra* was the only species in this study that did not have ornamentation on the eggshell surface (Fig. 5f). *Aonchotheca baylisi* had the smallest egg in the genus (44.75–50.14 × 24.92–28.97 μm), in contrast with *A. myoxinitelae* (55.44–61.57 × 24.77–26.76 μm).

### *Genus* Baruscapillaria

A total of five species were collected from the collections of MNHN and FIOCRUZ. The hosts of all the species were registered as avian: *Baruscapillaria obsignata* in *Gallus gallus domesticus*; *B. rudolphi* in *Tinamus solitarius*; *B. spiculata* in *Carbo vigua*; *B. falconis* in *Tyto alba*; *B. resecta* in *Garrulus glandariu*. RTI (Fig. 5f, r) and II (Fig. 5b, n) were observed in two species, each type, and one punctuated (Fig. 5j). The eggs were very similar within the genus in shape and in plug base morphology. *Baruscapillaria resecta* was the species with the biggest egg measurements (65.47–70.39 × 29.58–31.81 μm).

### *Genus* Capillaria

Four species were collected from both MNHN and FIOCRUZ. The hosts were registered as avian and mammal: *Capillaria venusta* in *Ramphasto toco*; *C. collaris* in *Gallus gallus domesticus*; *C. brasiliana* in *Nycticorax naevius*; *C. exigua* in *Erinaceus europaeus*. The morphologies of the eggs were very different in shape. The genus showed the three different types of ornamentations (RTI, RTII and punctuated).

### *Genus* Calodium

Only one species was collected from *Collection de Nématodes Zooparasites* of MNHN. The hosts were recorded as mammals: *Calodium hepaticum* in *Meriones persicus* and *Rattus rattus.* This species has a very peculiar morphology. The ornamentation is punctuated and, in a transversal view, a radial ornamentation is observed on the eggshell. The thickest eggshell was detected in this species (5.54 μm).

### *Genus* Echinocoleus

Two species were collected from the helminth collection of CHIOC/FIOCRUZ. The hosts registered were mammals: *Echinocoleus hydrochoeris* in *Hydrochoerus capybara*; *Ec. auritae* in *Metachirops opossum.* The ornaments identified were punctuated (Fig. 4d) and RTI (Fig. 4b), respectively. Both had a very thick eggshell (2.1–3.51 μm and 1.59–3.63 μm, respectively). *Echinocoleus auritae* has a particular eggshell ornament, with a prominent reticulated suface in the transversal view.

### *Genus* Eucoleus

Four species were collected from both collections. The hosts were avian and mammal: *Eucoleus perforans* in *Numida meleagris*; *E. annulatus* in *Gallus gallus domesticus*; *E. contortus* in *Sterna maxima* and *Ajaja ajaja*; *E. dubius* in *Attila cinereus*; *E. eberthi* in *Metachirops opossum*; *E. bacilatus* in *Apodemus sylvaticus*; *E. madjerdae* in *Mus musculus*; *E. dispar* in *Atlapetes semirufus*. Most eggs observed in the genus *Eucoleus* had the punctuated ornamentation (Fig. 3b, f, h, j, l, p), but one species had RTI and another species presented RTII. *Eucoleus* genus showed the most variable measurements of length (37.06–68.82 μm) and width (18.15–33.65 μm) among its species. The same was observed on plug base measurements, plug base length and width, and on eggshell thickness. The smallest of all capillariid species is *E. perforans* (37.06 × 18.91 μm), and the thinnest is *E. annulatus* (0.78 μm).

### *Genus* Pearsonema

Only one species was collected from *Collection de Nématodes Zooparasites* of MNHN. The host was registered as a mammal: *Pearsonema pulchra* in *Vulpes vulpes*. The egg had a very elongated morphology, with a prominent RTI eggshell.

### *Genus* Tridentocapillaria

Only one species was collected from the *Collection de Nématodes Zooparasites* of MNHN. The host was registered as avian: *Tridentocapillaria tridens* in *Cyanolanius madagascarinus*. The species *T. tridens* had RTII ornamentation.

### Discriminant analyses and artificial intelligence/machine learning approaches

**Fig. 6:**
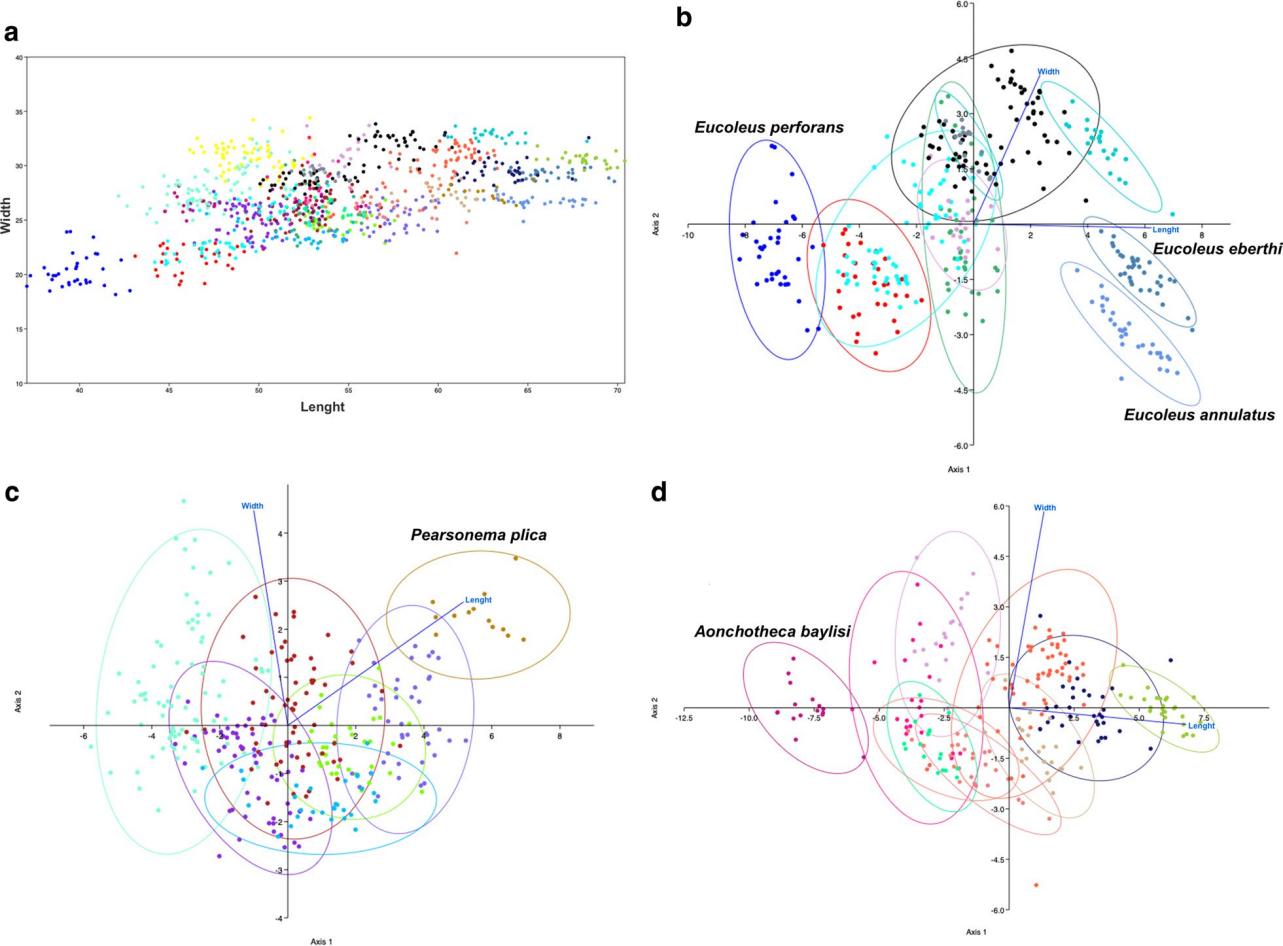
Discriminant analyses considering measures of length and width of capillariid eggs. Each color represents one species. **a** Plot of all specimens in the present study. **b**, **c**, **d** Plot of specimens according to the classification of ornamentation, **b** punctual, **c** reticulated type I and **d** reticulated type II

The graphic XY of length and width measures for all species revealed a strong superposition of data with a more discriminant distribution in the egg length than egg width parameter (Fig. 6a). The graphics of discriminant analysis by eggshell ornamentation showed the same pattern of species overlapping, with only one to three species groups showing adequate parameters for capillariid identification (Fig. 6b–d), with the discrimination of *E. perforans*, *E. annulatus*, *E. eberthi* (Punctuated) (Fig. 6b), *P. plica* (RTII) (Fig. 6c) and *A. baylisi* (RTII) (Fig. 6d).

The LMT algorithm showed the highest values in all metrics compared with the other algorithms (Table 2). However, the LMT algorithm does not return representations of traditional decision trees that could be representative of a taxonomic classification. The Majority Voting algorithm showed high metric values, but the combined classifiers did not attenuate the errors revealed in each algorithm alone (Table 2; Additional file 1: Tables S2, S3). From all the algorithms that produced representative decision trees, J48 showed higher values in all metrics, with the exception of AUC (0.979), which was higher for REPTree (0.986) in all parameter combinations (Table 2). The performance of algorithms using morphological and morphometric data without ecological parameters (MM) revealed the worst metrics (Table 2).

**Table 2:** Algorithms and parameters considered in the ML/IA analysis

Algorithms	MM + GL + H							
	Correct instances (%)	Kappa	Specificity	Sensitivity	AUC	Accuracy	NPV	PPV
J48	93.172	0.92	0.962	0.966	0.979	0.964	0.965	0.964
Random Tree	89.257	0.89	0.934	0.951	0.944	0.942	0.949	0.936
REPTree	90.963	0.90	0.959	0.945	0.986	0.952	0.943	0.961
LMT	96.385	0.96	0.981	0.982	0.999	0.981	0.981	0.982
Majority Voting	94.679	0.94	0.974	0.970	0.972	0.964	0.969	0.975

Algorithms	MM + H							
	Correct instances (%)	Kappa	Specificity	Sensitivity	AUC	Accuracy	NPV	PPV
J48	88.253	0.88	0.932	0.941	0.955	0.937	0.939	0.934
Random Tree	86.646	0.86	0.949	0.935	0.93	0.942	0.933	0.950
REPTree	85.943	0.85	0.919	0.911	0.979	0.915	0.908	0.921
LMT	93.975	0.94	0.965	0.972	0.998	0.968	0.971	0.966
Majority Voting	91.867	0.91	0.955	0.959	0.957	0.957	0.958	0.956

Algorithms	MM + GL							
	Correct instances (%)	Kappa	Specificity	Sensitivity	AUC	Accuracy	NPV	PPV
J48	92.570	0.92	0.960	0.961	0.975	0.961	0.960	0.961
Random Tree	91.566	0.91	0.951	0.959	0.956	0.955	0.958	0.953
REPTree	89.056	0.88	0.942	0.941	0.98	0.941	0.939	0.944
LMT	96.686	0.96	0.984	0.982	0.999	0.983	0.981	0.985
Majority Voting	95.683	0.95	0.980	0.975	0.978	0.978	0.975	0.980

Algorithms	MM							
	Correct instances (%)	Kappa	Specificity	Sensitivity	AUC	Accuracy	NPV	PPV
J48	84.538	0.84	0.918	0.912	0.912	0.915	0.909	0.920
Random Tree	82.228	0.81	0.885	0.917	0.917	0.901	0.915	0.888
REPTree	84.337	0.83	0.915	0.912	0.912	0.914	0.909	0.918
LMT	91.867	0.91	0.956	0.958	0.958	0.957	0.957	0.957
Majority Voting	89.056	0.88	0.934	0.949	0.949	0.941	0.947	0.936

Performance of algorithms is reported as specificity, sensitivity and accuracy following [24] and as corrected classified instances, kappa coefficient and AUC, as generated by Weka 3.8.3 software

*MM* morphological and morphometric data, *GL* geographical location, *H* host, *AUC* area under the receiver-operating characteristic (ROC) curve, *NPV* negative predictive value, *PPV* positive predictive value

The statistical test showed a highly significant difference among the algorithms (*P* < 0.001) and among the parameters (*P* < 0.001), thus rejecting the null hypothesis of equal proportions (Additional file 1: Tables S2). The Marascuilo results between the combination of parameters showed statistical differences when no ecological parameters were applied (MM), using all the algorithms, except in RandomTree and Majority Voting. Comparing algorithms, a significant difference is observed when the LMT is applied for all the parameters, except for the MM parameter compared with Majority Voting (Additional file 1: Tables S2). In general, there was no statistical significance when comparing the Majority Voting algorithm with each algorithm that produced representative decision trees (J48, RandomTree and REPTree). Excluding LMT and Majority Voting (no representative decision trees) and also the MM parameter (lowest AUC values), no statistical significance was seen between REPTree and J48 in all parameter combinations (Additional file 1: Tables S2). We chose the J48 algorithm using all MM + H + GL parameters (higher AUC values) for decision tree representation. However, there was no significant difference when compared with MM + GL parameters but there was a difference compared with the MM + H parameters.

**Fig. 7:**
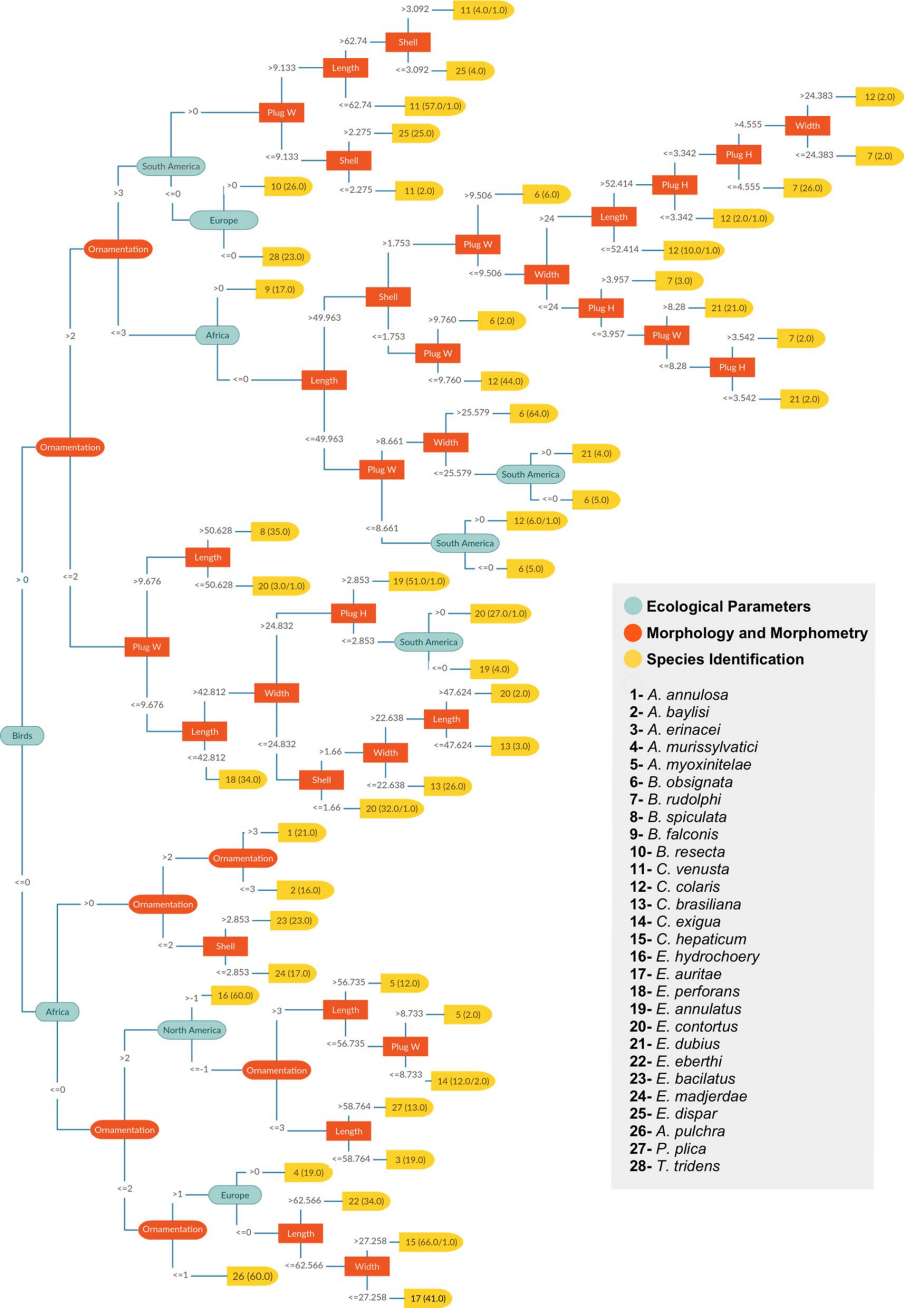
Decision tree for Capillaridae species discrimination using the J48 algorithm with MM + GL + H parameters. MM: includes the attributes: length, width, plug base width, plug base height, shell thickness and ornamentation type (in orange). GL: geographic location includes North America, Central America, South America, Europe, Africa, Asia and Oceania (in green). H: includes fish, amphibians, reptiles, avians and mammals (in green). Ornamentation 1: smooth; 2: punctuated; 3: reticulated type I; 4: reticulated type II. Numbers in yellow are the capillariid species and in parentheses are correct/incorrect entries by the program. The numbers in the lines between the parameters represent the range of values considered to identify a specimen. Generated by Weka 3.8.3 software

Figure 7 presents the decision trees generated by the J48 algorithm, applying all ecological parameters and morphological and morphometric data. The decision trees constructed using morphological and morphometric data plus only host (MM + H) are available in the supporting information (Additional file 2: Fig. S1). The same is found for MM + GL (Additional file 2: Fig. S2) and morphological and morphometric data only (Additional file 2: Figs S3, S4), considering three different ornamentation types, punctuated (Additional file 2: Fig. S3), RTI (Additional file 2: Fig. S4) and RTII (Additional file 2: Fig. S5). A classic taxonomic key was made for comparison, using the decision tree generated on AI program Weka 3.8.3 software (Additional file 2: Fig. S6).

## Discussion

Numerous species of capillariids have low host specificity, for instance, *Paracapillaria phillipinensis* is the only one known to parasitize two different classes of vertebrates, mammals and birds [26]. The difference in natural hosts could imply variability in the shape and/or size of eggs as phenotypical plasticity. This phenomenon occurs when the same species infects different hosts and presents different parasite phenotypes [27], as previously reported in *Schistosoma mansoni* adult worms. As observed in adult worms, the phenomenon can occur in other development stages, such as eggs.

Initially, to classify capillariids Romashov divided their eggs into six groups, considering only the eggshell surface ornaments and the site of parasite infection. All capillariids analyzed were from mammals, and the author concluded that a relation between those variables is enough to determine the genus, relatively unmistakably [8]. However, in coprological surveys and paleoparasitological studies it is impossible to define the site of infection, because the only datum recorded is the egg itself, sometimes also the host [6, 28].

In the present study, the punctuated ornamentation (six species) is predominant in the genus *Eucoleus* (Table 1). Although *E. dispar* has a different ornamentation from the other seven species described here, it is similar to *E. aerophilus*, as seen in the literature [29]. This is supported by molecular phylogenetic analyses, showing a close relation between them [4, 6]. However, molecular information from other known species of the genus was unavailable. *Eucoleus dubius* is also in another category with RTI ornamentation.

In the genus *Aonchotheca* the RTII ornamentation predominates (Table 1). Although this ornament is observed in *Baruscapillaria*, *Capillaria*, *Eucoleus* and *Tridentocapillaria*, the frequency is not too high. The genus *Echinocoleus* exhibited RTI and punctuated ornamentations, but this cannot be assumed to be a pattern, as the genus had only two species studied. *Pearsonema plica* and *C. collaris* presented RTI ornamentation, although the egg morphology was different; one was narrowed on the extremity (Fig. 4g) and the other was rounder (Fig. 5c), respectively.

Regarding the statistical analysis of egg measures, no relation between genera was detected. The same was observed for discriminating species. Even though length showed more relevance than width, a large part of the measures overlapped, and it was impossible to discriminate among most of them. When discriminant analysis was employed using the dataset separated by ornamentations, only 5 species among 28 could be identified (*E. perforans*, *E. annulatus*, *E. eberthi*, *P. plica* and *A. baylisi*). The results of discriminant analysis indicated the need to use a more robust tool that can integrate additional variables for species identification.

The ML/AI analysis revealed that when parameters related to geographical location and host were included, the reliability of the decision tree was higher with all algorithms used (Table 2). Although the LMT algorithm exhibited more reliable results, it did not produce a decision tree. Consequently, it is not functional in the biological sense and, more importantly, for application in future taxonomic identifications. The LMT algorithm would be useful if there was no need to understand how such taxonomic identification produced a specific classification.

Regarding ecological parameters, the H parameter may be more robust because, except for two genera (*Capillaria* and *Eucoleus*), it was possible to employ the taxonomic level of class and use one H entrance, avoiding decision errors. Regarding GL, first, for a more complete dataset, the parameter was defined by all the continents where the species was recorded, based on an extensive literature revision of capillariid identifications. However, species with worldwide distribution, such as *C. hepaticum*, presented multiple re-entrances in the dataset. The observed ML decision errors indicate that, whenever such worldwide distribution exists, it will be necessary to have additional egg features to improve the results. When using two different entrances for the same specimens, both for H and GL, the program tends to choose which one differentiates more between species. This could be erroneous because it does not consider the second entrance as a possible variable. For this reason, the GL parameter was then expressed as the site where specimens were collected, based on the FIOCRUZ and MNHN files. Therefore, the information on species distribution, used for geographical location, is restricted.

Although 12 decision trees were produced, as shown in Table 2, the trees generated by MM + GL + H exhibited the highest metric values in all algorithms (Fig. 7), with the exception of RandomTree where MM + GL displayed higher performance on most metric values. In general, GL showed the most relevant parameter in the presence of H. These results revealed the relevance of ecological characteristics of specimens for the species discrimination. However, geographical location showed better results in all parameters compared to the host. No significant difference was shown between them (MM + H or MM + GL), which means one could have compensated the absence of the other in those data. Otherwise, for the J48 algorithm, the Marascuilo test showed that the host had a significant difference from all parameters included, giving the notion that the GL is more reliable than H. Moreover, we did not only consider the tree with MM as it had the worst metric performance.

Out of the three algorithms that produced a traditional decision tree, REPTree had the highest AUC value (0.986) and was statistically different from RandomTree (0.956), but not from J48 (0.979). Both REPTree and J48 algorithms were tested statistically with all the parameter combinations to see if we could find the best representation tree among them. In J48 MM + GL + H was statistically different from MM + H. Additionally, J48 had all the other metric values higher than REPTree (Table 2), and the parameters MM + GL + H had higher values than the other combinations, which affected our decision on representing the decision tree with J48 with all parameters.

One way to improve the result of the classifiers is to make a Majority Vote; as a result, the class with the highest number of votes is valid. There is a strong premise in this approach: it is assumed that voting entities will not err for the same classifications; in many situations, this can be assumed as true. However, when this approach was applied to the problem, we observed a drop in performance in relation to LMT, the best algorithm. This is possible because the expectation of the algorithms not to err for the same opinions has been frustrated, that is, the algorithms agree on their common mistakes. This might reflect the fact that the four algorithms belong to the same category of decision tree solvers. Thus, it makes sense that there is a possibility in this category for resolvers to induce some bias in agreeing to be correct in some cases and in agreeing to be wrong in other cases.

The Majority Voting algorithm, used to combine four algorithms, revealed higher metric values than J48, Random Tree and REPTree, with the exception of the AUC value, where REPTree (0.98) had a better performance. The LMT algorithm was the best algorithm in all metrics. Even though the Majority Voting does not add value to the analysis, it allows us to understand how J48, Random Tree and REPTree work. The fact that the Majority Voting had lower metric values than the LMT says that the contribution of the three other algorithms hinders the results, which suggests the three algorithms are wrong in the same cases. This makes them the majority, and the final decision becomes wrong. Therefore, while the LMT algorithm is right, the Majority Voting is wrong because of that wrong majority decision. It also cannot be used as a taxonomic key for the same reason as the LMT, discarding their application for the article’s purpose.

The ML/AI approach have been recently used to analyze the relationships among *Strongyloides* genotypes using multi-locus sequence typing, considering hosts and geographic distribution. This analysis showed the presence of different populations that were not evident using smaller datasets [13], corroborating the importance of a bigger dataset and the use of ML/AI in the classification of helminths.

The present study has some limitations relating to the dataset. It contains 28 species and 8 genera of capillariids out of more than 300 species and 25 genera described. Therefore, it contributes with a small portion of the real scenario of the biological diversity in capillariids. Despite about 30 eggs each examined, some species are represented by one specimen, what could be a restriction in the possible intraspecific and ecological variations. In addition, multiple hosts or geographical origins in the same species could be interpreted by the system as a discrepant character and, consequently, the learning is wrongly addressed. However, capillariid species in general are not so restricted. The solution we found was both a generalization and constriction of information on host and geographical location, respectively. The addition of new curated information from other biological helminth collections will enable the construction of a stronger, well-supported dataset and a better taxonomic definition using ML/AI. To our knowledge, this study is the first to apply artificial intelligence techniques to the taxonomic definition of biological species, opening an opportunity of application in health, biodiversity and technology research in other important taxa.

## Conclusions

The machine learning/artificial intelligence approach presented herein is an initial methodology for parasite species identification using capillariids as a model. The present study makes available a solid representation of capillariids deposited in two large and diverse institutional collections of the world, CHIOC/FIOCRUZ and Collection de Nématodes Zooparasites/MNHN. It supports the identification of capillariids with the characterization of 28 species and 8 genera, generating a catalog for future references. Furthermore, it supplies new data in the characterization of nematode eggs, a field that lacks knowledge in parasite morphological description, which comprises ecological and health surveys, as well as paleoparasitological research. Other collections can apply the same ML/AI methodologies proposed here and increase the species and families described.

## Supplementary Information

**Additional file 1: Table S1.** Morphometry: measurements of length, width, plug width, plug thickness and shell thickness; morphology: shell ornamentations, 1, smooth; 2, punctuated; 3, reticulated type I; 4, reticulated type II; host: fish, amphibian, reptile, avian, mammals; geographical location: South America, Central America, North America, Europe, Africa, Asia, Oceania. Response variable were 1 = yes or presence, 0 = no or absence, and -1 = no information. **Table S2.** Results of statistical analysis using AUC values performed on RStudio version 3.5.1. P-value of the Chi-square statistic among algorithms and combination of parameters applied. Marascuilo test results on the comparison of combination of parameters (MM + GL, MM + H, MM + GL + H) per algorithm (J48, Random Tree, REPTree, LMT and Majority Voting) and on the comparison of algorithms per combination of parameters applied. **Table S3.** Confusion Matrices generated by Weka 3.8.3 software showing the false positives, false negatives and true negatives resulted from each algorithm (J48, REPTree, Random Tree, LMT and Majority Voting) of each combination of parameters (MM + GL + H, MM + GL, MM + H and MM).

**Additional file 2: Figure S1.** Decision trees generated by Weka software using the J48 algorithm, including trees with the parameter MM + GL (Fig. 1), MM + H (Fig. 7), MM for P ornamentation (Fig. 2), MM for RTI ornamentation (Fig. 3) and MM for RTII ornamentation (Fig. 4). Generated by Weka 3.8.3 software. **Figure S2.** Taxonomic Key of eggs of Capillariidae created from the tree generated by Weka using the J48 algorithm and MM + GL + H parameters.

## Data Availability

All data generated or analysed during the present study are included in this article and its additional files.

## Abbreviations

ML: Machine learning
CHIOC: Coleção Helmintológica do Instituto Oswaldo Cruz
IOC: Instituto Oswaldo Cruz
FIOCRUZ: Fundação Oswaldo Cruz
MNHN: Muséum National d’Histoire Naturelle de Paris
MM: Morphological and morphometric values
GL: Geographical location
H: Host
LMT: Logistic model trees
AI: Artificial intelligence
RTI: Reticulated type I
RTII: Reticulated type II
AUC: Area under the receiver-operating characteristic curve
ROC: Receiver-operating characteristic
NPV: Negative predictive value
PPV: Positive predictive value
