# Supplementary material for: Machine learning approach to support taxonomic species discrimination based on helminth collections data
Source: Parasit Vectors. 2021 May 1;14:230. doi: 10.1186/s13071-021-04721-6 (PMC8088700; doi:10.1186/s13071-021-04721-6)
Supplement: Supplementary file 2 — Additional file 2: Figure S1. Decision trees generated by Weka software using the J48 algorithm, including trees with the parameter MM + GL (Fig. 1), MM + H (Fig. 7), MM for P ornamentation (Fig. 2), MM for RTI ornamentation (Fig. 3) and MM for RTII ornamentation (Fig. 4). Generated by Weka 3.8.3 software. Figure S2. Taxonomic Key of eggs of Capillariidae created from the tree generated by Weka using the J48 algorithm and MM + GL + H parameters. [file 13071_2021_4721_MOESM2_ESM.docx]

**Additional file 2: Figure S1.** Decision trees generated by Weka software using the J48 algorithm, including trees with the parameter MM+GL (Fig. 1), MM+H (Fig. 2), MM for P ornamentation (Fig. 3), MM for RTI ornamentation (Fig. 4) and MM for RTII ornamentation (Fig. 5). Generated by Weka 3.8.3 software.


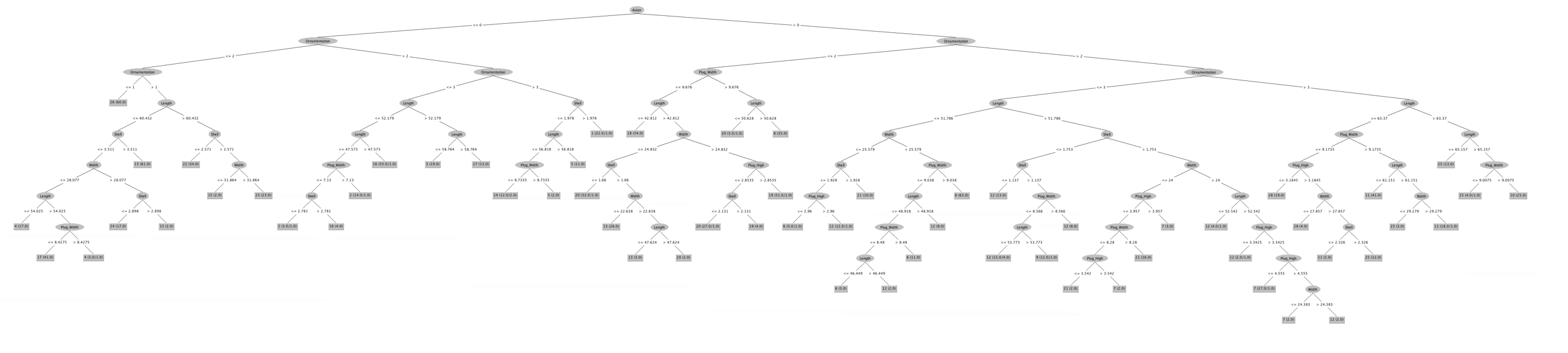


**Fig. 1. Decision tree generated by Weka software using J48 algorithm with MM plus only Host parameters that include the attributes;** for MM: length, width, plug base width, plug base height, shell thickness; and for Host: fish, amphibian, reptile, avian and mammals. Generated by Weka 3.8.3 software.


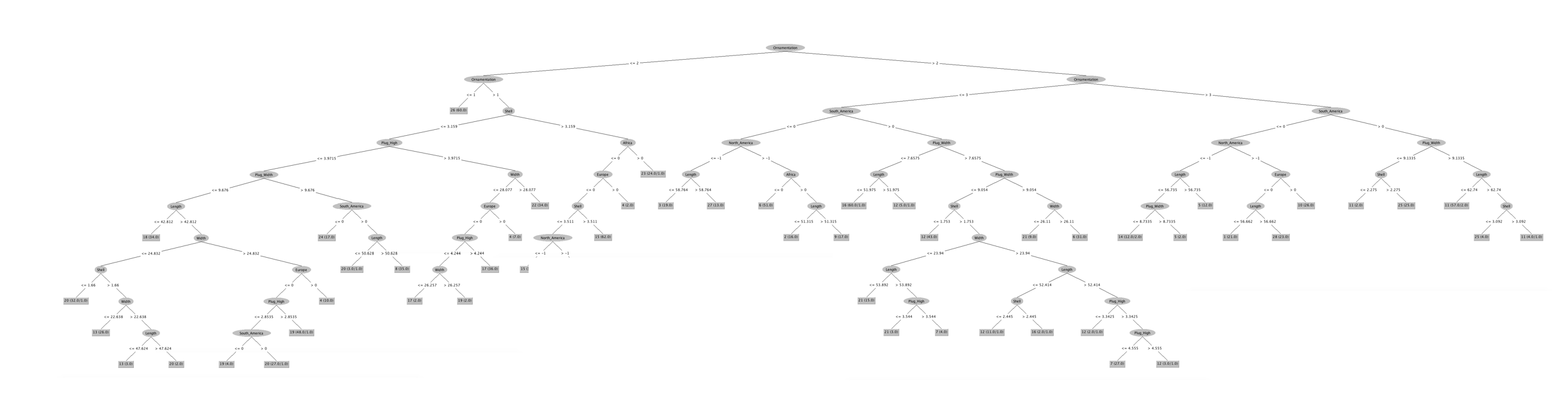


**Fig. 2. Decision tree generated by Weka software using J48 algorithm with only MM plus only geographical location parameters that include the attributes;** for MM: length, width, plug base width, plug base height, shell thickness; and for geographical location: South America, Central America, North America, Europe, Africa, Asia, Oceania. Generated by Weka 3.8.3 software.


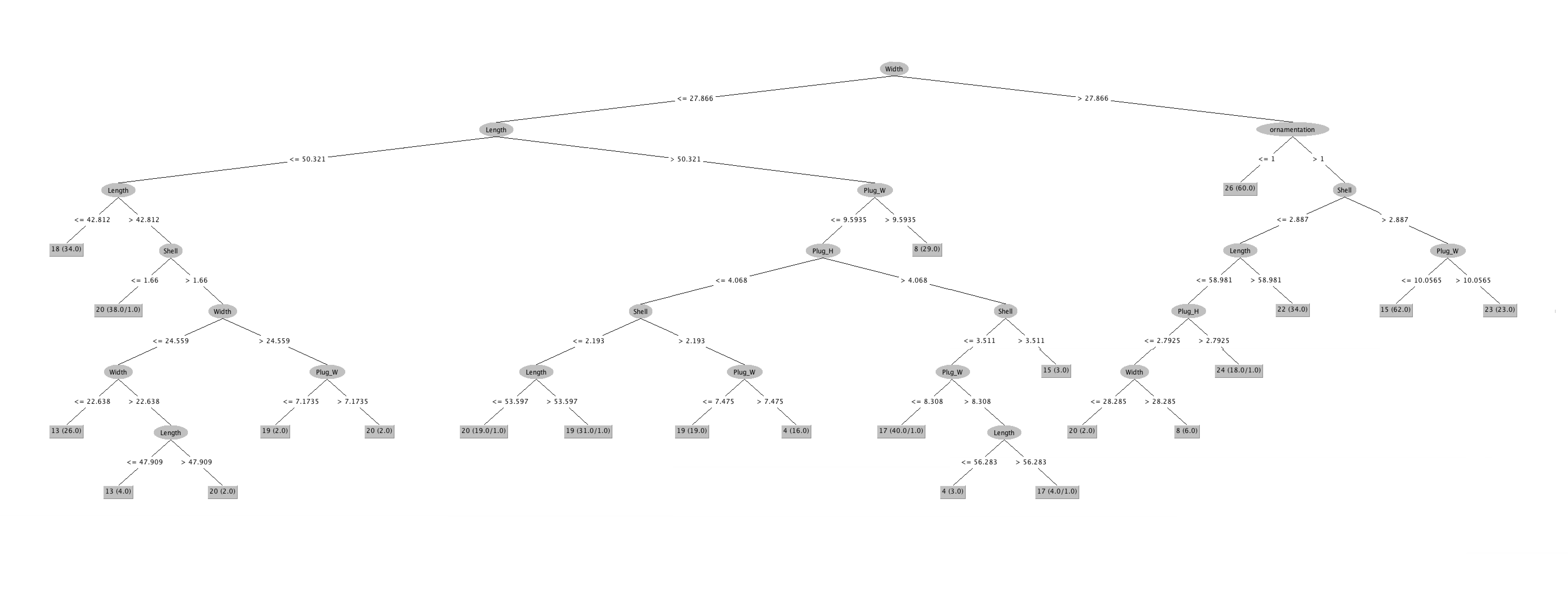


**Fig. 3. Decision tree generated by Weka software using J48 algorithm with only MM parameters that include the attributes: length, width, plug base width, plug base height, shell thickness, for punctuated ornamentation**. Generated by Weka 3.8.3 software.


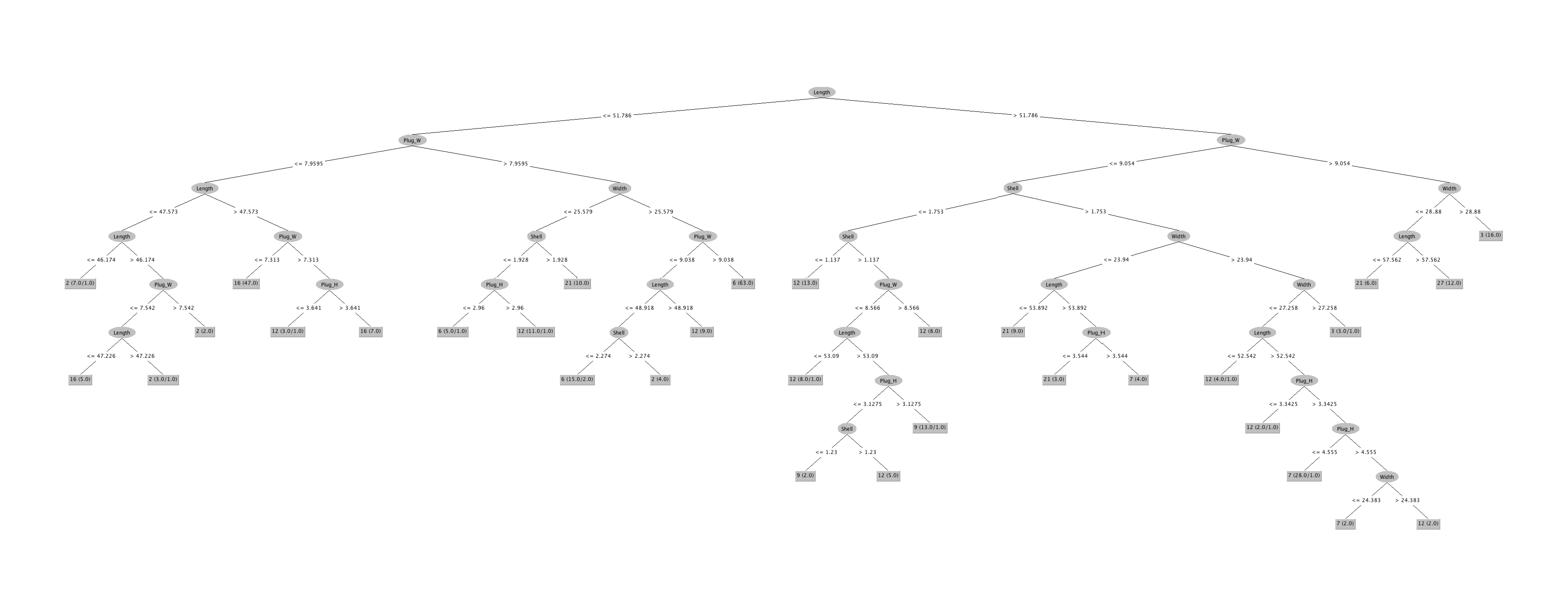


**Fig. 4. Decision tree generated by Weka software using J48 algorithm with only MM parameters that include the attributes: length, width, plug base width, plug base height, shell thickness, for reticulated type I ornamentation.** Generated by Weka 3.8.3 software.


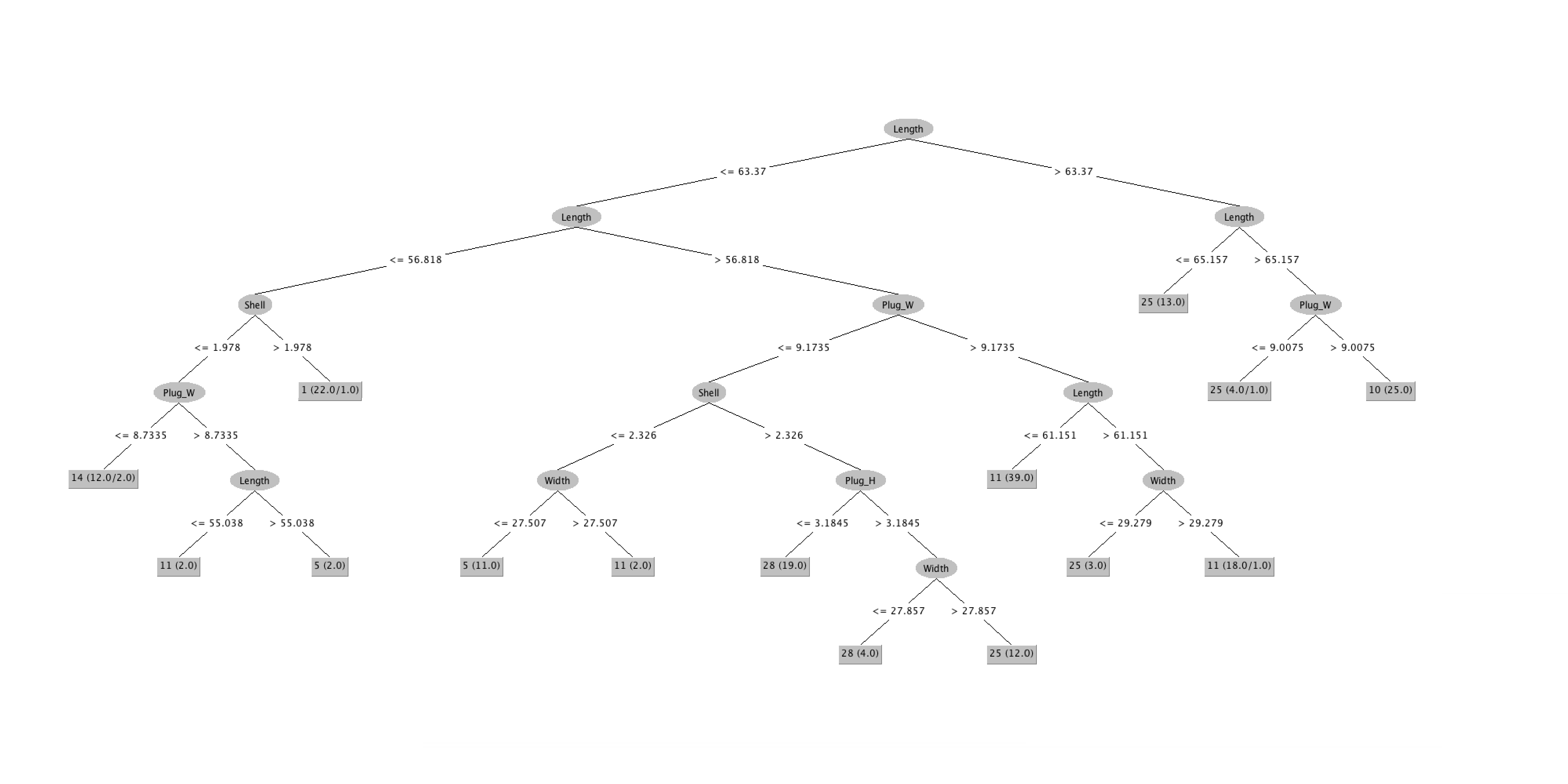


**Fig. 5. Decision tree generated by Weka software using J48 algorithm with only MM parameters that include the attributes: length, width, plug base width, plug base height, shell thickness, for reticulated type II ornamentation.** Generated by Weka 3.8.3 software.

**Figure S2.** Taxonomic Key of eggs of Capillariidae created from the tree generated by Weka using the J48 algorithm and MM+GL+H parameters.

1a. Avian Host go to 2

1b. Mammalian Host go to 32

2a. Reticulated Ornamentation go to 3

2b. Punctuated Ornamentation go to 25

3a. Reticulated Type 2 Ornamentation go to 4

3b. Reticulated Type 1 Ornamentation go to 9

4a. Geographical Location: South America go to 5

4b. Geographical Location: Europe *Baruscapilaria resecta*

4c. Geographical Location: Africa *Tridentocapillaria tridens*

5a. Plug Base Width LARGER than 9.1335μm go to 6

5b. Plug Base Width LESSER than 9.1335μm go to 8

6a. Egg Total Length LARGER than 62.74μm go to 7

6b. Egg Total Length LESSER than 62.74μm *Capillaria venusta**

7a. Eggshel Thickness LARGER than 3.092μm *Capillaria venusta**

7b. Eggshel Thickness LESSER than 3.092μm *Eucoleus dispar**

8a. Eggshel Thickness LARGER than 2.275μm *Eucoleus dispar**

8b. Eggshel Thickness LESSER than 2.275μm *Capillaria venusta**

9a. Geographical Location: Africa *Baruscapillaria falconis*

9b. Geographical Location: Outside Africa go to 10

10a. Egg Total Length LARGER than 49.963μm go to 11

10b. Egg Total Length LESSER than 49.963μm go to 21

11a. Eggshel Thickness LARGER than 1.753μm go to 12

11b. Eggshel Thickness LESSER than 1.753μm go to 20

12a. Plug Base Width LARGER than 9.5065μm *Baruscapillaria obsignata**

12b. Plug Base Width LESSER than 9.5065μm go to 13

13a. Egg Total Width LARGER than 24μm go to 14

13b. Egg Total Width LESSER than 24μm go to 17

14a. Egg Total Length LARGER than 52.414μm go to 15

14b. Egg Total Length LESSER than 52.414μm *Capillaria collaris**

15a. Plug Base Height LARGER than 4.555μm go to 16

15b. Plug Base Height BETWEEN 3.3425μm and 4.555μm *Baruscapillaria Rudolphi**

15c. Plug Base Height LESSER than 3.3425μm *Capillaria collaris**

16a. Egg Total Width LARGER than 24.383μm *Capillaria collaris**

16b. Egg Total Width LESSER 24.383μm *Baruscapillaria Rudolphi**

17a. Plug Base Height LARGER than 3.975μm *Baruscapillaria Rudolphi**

17c. Plug Base Height LESSER than 3.975μm go to 18

18a. Plug Base Width LARGER than 8.28μm *Eucoleus dubius**

18b. Plug Base Width LESSER than 8.28μm go to 19

19a. Plug Base Height LARGER than 3.542μm *Baruscapillaria rudolphi**

19c. Plug Base Height LESSER than 3.542μm *Eucoleus dubius**

20a. Plug Base Width LARGER than 9.7605μm *Baruscapillaria obsignata**

20b. Plug Base Width LESSER than 9.7605μm *Capillaria collaris**

21a. Plug Base Width LARGER than 8.661μm go to 22

21b. Plug Base Width LESSER than 8.661μm go to 24

22a. Egg Total Width LARGER than 25.579μm *Baruscapillaria obsignata**

22b. Egg Total Width LESSER 25.579μm go to 23

23a. Geographical Location: South America *Eucoleus dubius**

23b. Geographical Location: Central America *Baruscapillaria obsignata**

24a. Geographical Location: South America *Capillaria collaris**

24b. Geographical Location: Central America *Baruscapillaria obsignata**

25a. Plug Base Width LARGER than 9.676μm go to 26

25b. Plug Base Width LESSER than 9.676μm go to 27

26a. Egg Total Length LARGER than 50.628μm *Baruscapillaria spiculata*

26b. Egg Total Length LESSER than 50.628μm *Eucoleus contortus**

27a. Egg total Length LARGER than 42.812μm go to 28

27b. Egg total Length LESSER than 42.812μm *Eucoleus perforans*

28a. Egg Total Lenght LARGER than 57.743μm *Eucoleus annulatus*

28b. Egg Total Lenght LESSER 57.743μm go to 29

29a. Eggshel Thickness LARGER than 1.66μm go to 30

29b. Eggshel Thickness LESSER than 1.66μm *Eucoleus contortus**

30a. Egg Total Width LARGER than 22.638μm go to 31

30b. Egg Total Width LESSER than 22.638μm *Capillaria brasiliana**

31a. Egg Total Length LARGER than 47.624μm *Eucoleus contortus**

31b. Egg Total Length LESSER than 47.624μm *Capillaria brasiliana**

32a. Geographical Location: Africa go to 33

32b. Geographical Location: Outside Africa go to 35

33a. Punctuated Ornamentation go to 34

33b. Reticulated Type 1 Ornamentation *Aonchotheca baylisi*

33c. Reticulated Type 2 Ornamentation *Aonchotheca annulosa*

34a. Eggshel Thickness LARGER than 2.853μm *Eucoleus bacilatus*

34b. Eggshel Thickness LESSER than 2.853μm *Eucoleus madjerdae*

35a. Smooth Ornamentation *Aonchotheca pulchra*

35b. Punctuated Ornamentation go to 41

35c. Reticulated Ornamentation go to 36

36a. Geographical Location: North America *Echinocholeus hydrochoeri*

36b. Geographical Location: Outside North America go to 37

37b. Reticulated Type 1 Ornamentation go to 38

37c. Reticulated Type 2 Ornamentation go to 39

38a. Egg total Length LARGER than 58.764μm *Pearsonema plica*

38b. Egg total Length LESSER than 58.764μm *Aonchotheca erinacei*

39a. Egg total Length LARGER than 56.735μm *Aonchotheca myoxinitelae**

39b. Egg total Length LESSER than 56.735μm go to 40

40a. Plug Base Width LARGER than 8.7335μm *Aonchotheca myoxinitelae**

40b. Plug Base Width LESSER than 8.7335μm *Capillaria exigua*

41a. Geographical Location: Europe *Aonchotheca murissylvatici*

41b. Geographical Location: Outside Europe go to 42

42a. Egg total Length LARGER than 62.566μm *Eucoleus eberthi*

42b. Egg total Length LESSER than 62.566μm go to 43

43a. Egg Total Width LARGER than 27.258μm *Calodium hepaticum*

43b. Egg Total Width LESSER than 27.258μm *Echinocoleus auritae*

Obs.: The species with * have more than one way of identification in the taxonomic key.
